# A Genome-Wide Association Study of Genetic Variants of Apolipoprotein A1 Levels and Their Association with Vitamin D in Korean Cohorts

**DOI:** 10.3390/genes13091553

**Published:** 2022-08-29

**Authors:** Young Lee, Ji Won Yoon, Ye An Kim, Hyuk Jin Choi, Byung Woo Yoon, Je Hyun Seo

**Affiliations:** 1 Veterans Medical Research Institute, Veterans Health Service Medical Center, Seoul 05368, Korea; 2 Department of Applied Statistics, Chung-Ang University, Seoul 06974, Korea; 3 Healthcare System Gangnam Center, Seoul National University Hospital, Seoul 06236, Korea; 4 Division of Endocrinology, Department of Internal Medicine, Veterans Health Service Medical Center, Seoul 05368, Korea; 5 Department of Internal Medicine, College of Medicine, Chung-Ang University, Seoul 06974, Korea

**Keywords:** apolipoprotein A1, genome-wide association study, cardiovascular disease, single nucleotide polymorphisms, vitamin D

## Abstract

Dyslipidemia is an important independent risk factor for cardiovascular disease (CVD). Specifically, apolipoprotein A1 (ApoA1), apolipoprotein B (ApoB), and the ApoB/A1 ratio have been linked to CVD. We conducted a genome-wide association study meta-analysis of two Korean cohorts containing a total of 12,924 patients to identify novel single nucleotide polymorphisms (SNPs) associated with ApoA1 and ApoB levels and the ApoB/A1 ratio. Additionally, an expression quantitative trait locus (eQTL) and differentially expressed genes (DEGs) analysis were performed. The statistically significant eQTL, DEG, and Gene Ontology (GO) results were used to explore the predicted interaction networks and retrieve the interacting genes and proteins. We identified three novel SNPs (rs11066280, *p* = 3.46 $\times \;10^{-21}$; rs1227162, *p* = 2.98 $\times \;10^{-15}$; rs73216931, *p* = 5.62 $\times \;10^{-9}$) associated with ApoA1. SNP rs73216931 was an eQTL for *KMT5A* in the pancreas and whole blood. The network analysis revealed that *HECTD4* and *MYL2:LINC1405* are associated with *AKT1*. Our in silico analysis of ApoA1 genetic variants revealed heart muscle-related signals. ApoA1 also correlated positively with vitamin D, and genes associated with ApoA1 and vitamin D were found. Our data imply that more research into ApoA1 is needed to understand the links between dyslipidemia and CVD and vitamin D and CVD.

## 1. Introduction

Globally, cardiovascular disease (CVD) is the leading cause of mortality, claiming an estimated 17.9 million lives annually [1]. It is widely accepted that dyslipidemia contributes to CVD [2,3], and lipoprotein levels are an independent risk factor for arteriosclerosis and CVD [4]. Apolipoprotein A1 (ApoA1) is a crucial functional component of high-density lipoprotein (HDL) particles, which reverse cholesterol transport from peripheral tissue to the liver [5], and ApoA1 decreases blood cholesterol levels and prevents atherosclerosis formation [6]. Apolipoprotein B (ApoB) is the main lipoprotein in low-density lipoprotein (LDL) cholesterol. Therefore, ApoA1 and ApoB analytical investigations are valuable in studying disorders such as CVDs and metabolic disease. In addition, the ApoB/ApoA1 ratio is now widely used to predict the occurrence of CVDs and metabolic syndrome [7] because it is a composite index that comprehensively reflects the lipid metabolism balance [8,9,10].

Although several genome-wide association studies (GWASs) of lipid panels, such as total cholesterol, triglycerides, and apolipoproteins, have been undertaken [11,12,13,14,15,16,17], only a few single nucleotide polymorphisms (SNPs) for ApoA1 and ApoB levels and the ApoB/A1 ratio have been identified, leaving several loci to be studied. Lipoprotein (a) is a highly heritable trait (heritability ranged from 068 to 0.98), suggesting that genetic factors play a major role [18,19,20]. The genes reported to affect ApoA1 levels are *APOA1*, *APOC3*, *APOA1-AS*, and *SIK3*, and those reported to affect ApoB levels are *APOB, GCK,* and *SORT1* [21]. Additionally, a recent study using Mendelian randomization (MR) for ApoB levels revealed numerous other candidate genes, including *CELSR2*, *ABCA6*, and *DOCK7* [22]. Since the ApoB/ApoA1 ratio was suggested as a clinical index for atherosclerosis, few GWASs have investigated the phenotypes associated with it. Vitamin D, a fat-soluble vitamin, plays an essential role in bone mineralization and calcium homeostasis [23], and a vitamin D deficiency is closely associated with CVD [24]. Because the metabolic process is linked to the production of both lipids and vitamin D [25], it should be instructive to examine the relationships among them at the genome level. Specifically, several genes in the GWAS Catalog, particularly *ABCA1*, *PDE3B*, *APOB*, and *APOA5*, were found to affect both vitamin D levels [26] and apolipoproteins. Therefore, we conducted a GWAS in a multi-cohort Korean population to find SNPs for ApoA1, ApoB, and ApoB/ApoA1.

## 2. Materials and Methods

### 2.1. Study Participants

**Figure 1:**
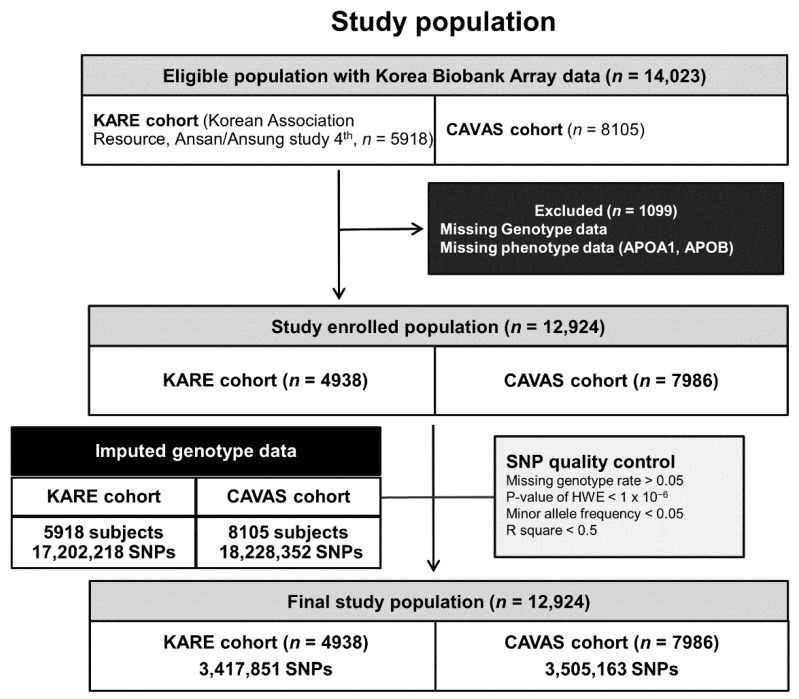
Schematic plot of study design: Meta-analysis of genome-wide association study data. HWE, Hardy–Weinberg Equilibrium.

Schematic plots of the analytical study design are provided in Figure 1. Data were obtained from two independent cohorts: The Korean Association Resource from Ansan and Ansung (KARE) cohort (*n* = 5918) and the Cardiovascular Disease Association Study (CAVAS, *n* = 8105) cohort. Each cohort has its own distinct characteristics. The KARE cohort is a representative cohort for genome research in Korea. It is a longitudinal cohort of people from the Ansan and Ansung communities in Korea gathered to study complex diseases, including diabetes. The CAVAS cohort is an elderly cohort of people from rural areas that includes many patients with CVDs. The exclusion criteria for this study were as follows: Missing genotype and phenotype data for ApoA1 and ApoB. Among the 14,023 participants in the 2 cohorts, 1099 were excluded, and 12,924 were enrolled (Figure 1). The institutional review boards of the Veterans Health Service Medical Center approved this study protocol (IRB No. 2020-03-009 and IRB No. 2019-08-014) with an informed consent waiver because our analyses were retrospective. This study was conducted in compliance with the Helsinki Declaration. The committee of the National Biobank of Korea approved the use of bioresources for this study (KBN-2019-054 and KBN-2020-071).

### 2.2. Biochemical Measurements

Serum levels of ApoA1 and ApoB were measured by immunoturbidimetric assay using a Roche-Hitachi Cobas 8000 c702 (Roche Diagnostics System, Switzerland) for both the KARE and CAVAS cohorts. Serum 25(OH)D levels were measured by a chemiluminescent microparticle immunoassay using an Architect i2000SR system (Abbott, Singapore) for the KARE and CAVAS cohorts.

### 2.3. Genotyping

We used a genome-wide set of variants genotyped with the Affymetrix Genome-Wide Human SNP Array 5.0 for the KARE cohort (*n* = 5918) and the Korea Biobank arrays (KoreanChip) for the CAVAS cohort (*n* = 8105). The KoreanChip was developed by the Center for Genome Science at the Korea National Institutes of Health using the Affymetrix Axiom^®^ Array (Affymetrix, Santa Clara, CA, USA). The KARE and CAVAS cohorts have already been described in further detail [27,28]. We used SHAPEIT2 (with the default setting of 2 Mb windows and 100 states) and IMPUTE2 (with chunk sizes of 5 Mb and the command option “–Ne 20000”) for haplotype phasing and imputation, respectively [29,30]. For imputation, the 1000 Genomes Phase III data were used as a reference panel. Any variant with genotype call rates under 95%, minor allele frequency (MAF) values below 0.05, or in violation of the Hardy–Weinberg equilibrium (*p* < 1 × $10^{-6}$) was removed. Only SNPs with quality scores greater than 0.5 were kept, resulting in 3,417,851 SNPs for the KARE cohort and 3,505,163 SNPs for the CAVAS cohort (Figure 1). The National Center for Biotechnology Information Human Genome Build 37 (hg19) was used to confirm gene locations.

### 2.4. Statistical Analyses

The baseline characteristics of the study population are presented as means with standard deviations for continuous variables and numbers with proportions for categorical variables. Association analysis between vitamin D and apolipoproteins (ApoA1, ApoB, ApoB/ApoA1) was conducted because vitamin D is associated with CVDs [24]. In addition, gene loci in the GWAS Catalog (https://www.ebi.ac.uk/gwas/ (accessed on 27 June 2022)) [31] associated with both vitamin D levels (including the Korean cohorts study [25]) and apolipoprotein levels were investigated and identified in our study.

Genome-wide association analyses for serum ApoA1, ApoB, and ApoB/ApoA1 were conducted using linear regression with an additive model. PLINK 1.9 was used within each cohort. Age, sex, body mass index, and ten principal component scores were included as covariates. Meta-analyses of the KARE and CAVAS cohorts were performed using METAL software (http://csg.sph.umich.edu/abecasis/meta (accessed on 19 April 2022)). Cochran’s Q-test for heterogeneity was conducted. Its *p*-value is marked with ‘*HetPVal’* [32], where *HetPVal* < 0.05 indicates heterogeneity between the two datasets [33]. The genomic inflation factor, λ, was calculated on the whole genome level by dividing the observed chi-squared test statistics by the expected chi-squared distribution median to ensure that no confounding was caused by population stratification in this study, close to 1 indicates no genetic inflation [34]. The regional plot of significant genetic variation was created using LocusZoom software [35]. The threshold for statistical significance in this model was *p* < 5.0 × $10^{-8}$, which is conventionally considered to reflect genome-wide significance.

### 2.5. Functional Annotation Analyses

Expression quantitative trait locus (eQTL) studies were performed using the Genotype-Tissue Expression dataset (https://gtexportal.org/home/ (accessed on 27 April 2022)), which contains a variety of densely genotyped data from donors to assess genetic variations within their genomes. Detailed methods, including normalization techniques, can be found at https://www.gtexportal.org/home/methods (accessed on 27 April 2022). The associated genes were further investigated for differentially expressed genes (DEGs) in human coronary artery cells treated with ApoA1 and phosphate-buffered saline controls using data from the Gene Expression Omnibus dataset (GSE53201) [36]. All array data are accessible from the Gene Expression Omnibus (GEO, http://www.ncbi.nlm.nih.gov/geo/ (accessed on 3 May 2022)) with the GSE53201 accession code. The GEO also provides GEO2R (http://www.ncbi.nlm.nih.gov/geo/geo2r/ (accessed on 3 May 2022)), an R-based web application that helps users analyze GEO data. Data after quantile normalization were used, and the Benjamini–Hochberg false discovery rate–adjusted 0.05 significance level was applied for multiple hypothesis test corrections [37]. Moreover, we annotated genes by constructing gene interaction networks with the STRING v.11 online platform (https://string-db.org/ (accessed on 29 June 2022)). The results for the corresponding biological processes, cellular components, and molecular functions from the Gene Ontology (GO) and Kyoto Encyclopedia of Genes and Genomes (KEGG) pathway enrichment analyses [38] were also identified from the STRING database.

## 3. Results

### 3.1. Characteristics of the Study Participants

A total of 14,023 eligible subjects were included in this study (5918 subjects from the KARE cohort and 8105 subjects from CAVAS). One thousand and ninety-nine subjects were excluded due to the exclusion criteria (Figure 1). Therefore, 12,924 participants (4938 subjects from the KARE cohort and 7986 from the CAVAS cohort) were analyzed in this study.

The mean age of the CAVAS cohort was higher than that of the KARE cohort (58.40 ± 8.82 years vs. 57.48 ± 8.46 years, *p* < 0.001, Table 1). There was no significant difference in body mass index (24.53 ± 3.02 kg/m^2^ in KARE vs. 24.54 ± 3.04 kg/m^2^ in CAVAS, *p =* 0.935) between the two cohorts. The proportions of systemic hypertension and diabetes in the CAVAS cohort were significantly higher than those in the KARE cohort (27% vs. 4.42%, *p* < 0.001, and 8.3% vs. 1.83%, *p* < 0.001, respectively). Total cholesterol (195.74 ± 34.29 mg/dL in KARE vs. 196.94 ± 35.32 mg/dL in CAVAS, *p =* 0.056) and LDL-cholesterol (124.23 ± 30.68 mg/dL in KARE vs. 123.71 ± 31.64 mg/dL in CAVAS, *p =* 0.36) did not differ significantly between the two cohorts, but the triglyceride and HDL-cholesterol levels in CAVAS were higher than those in KARE (*p* = 0.005, *p* < 0.001, respectively). In addition, the ApoA1 and ApoB levels in the CAVAS cohort were higher than those in KARE [(154.74 ± 26.64 mg/dL vs. 145.24 ± 24.52 mg/dL, *p* < 0.001) and (109.28 ± 25.24 mg/dL vs. 106.79 ± 24.26 mg/dL, *p* < 0.001), respectively].

### 3.2. GWAS Meta-Analysis of ApoA1, ApoB, and ApoB/ApoA1

**Figure 2:**
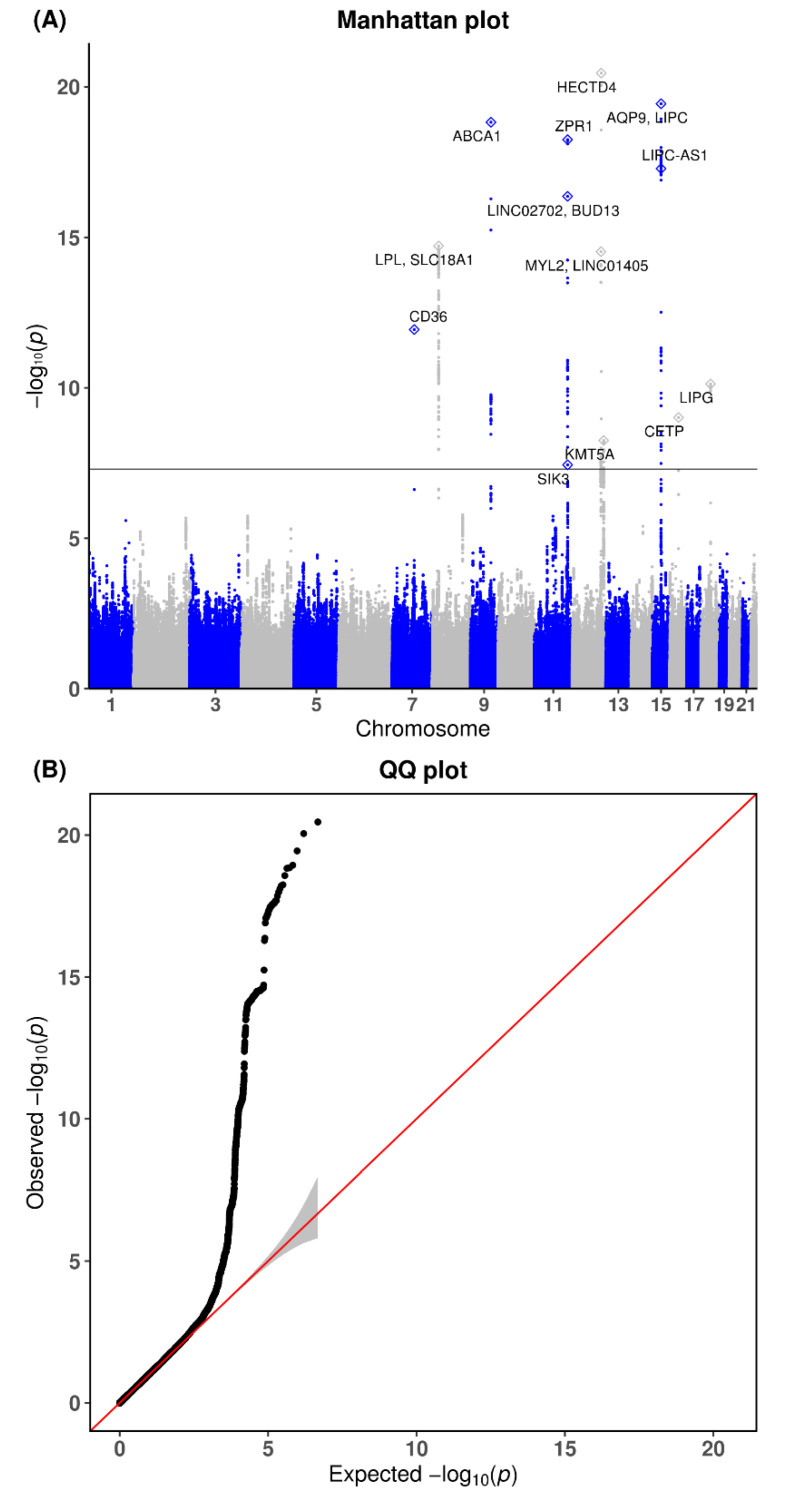
Genome-wide association of circulating ApoA1 by chromosome position, log10 *p*-value (Manhattan plot), and quantile–quantile plots for meta-analysis. (**A**) Manhattan plot of the *p*-values in the genome-wide association study meta-analysis for ApoA1. (**B**) Quantile-quantile (Q-Q) plot showing expected vs. observed values [−log10(*p*)values]. The expected line is shown in red, and confidence bands are shown in gray.

**Figure 3:**
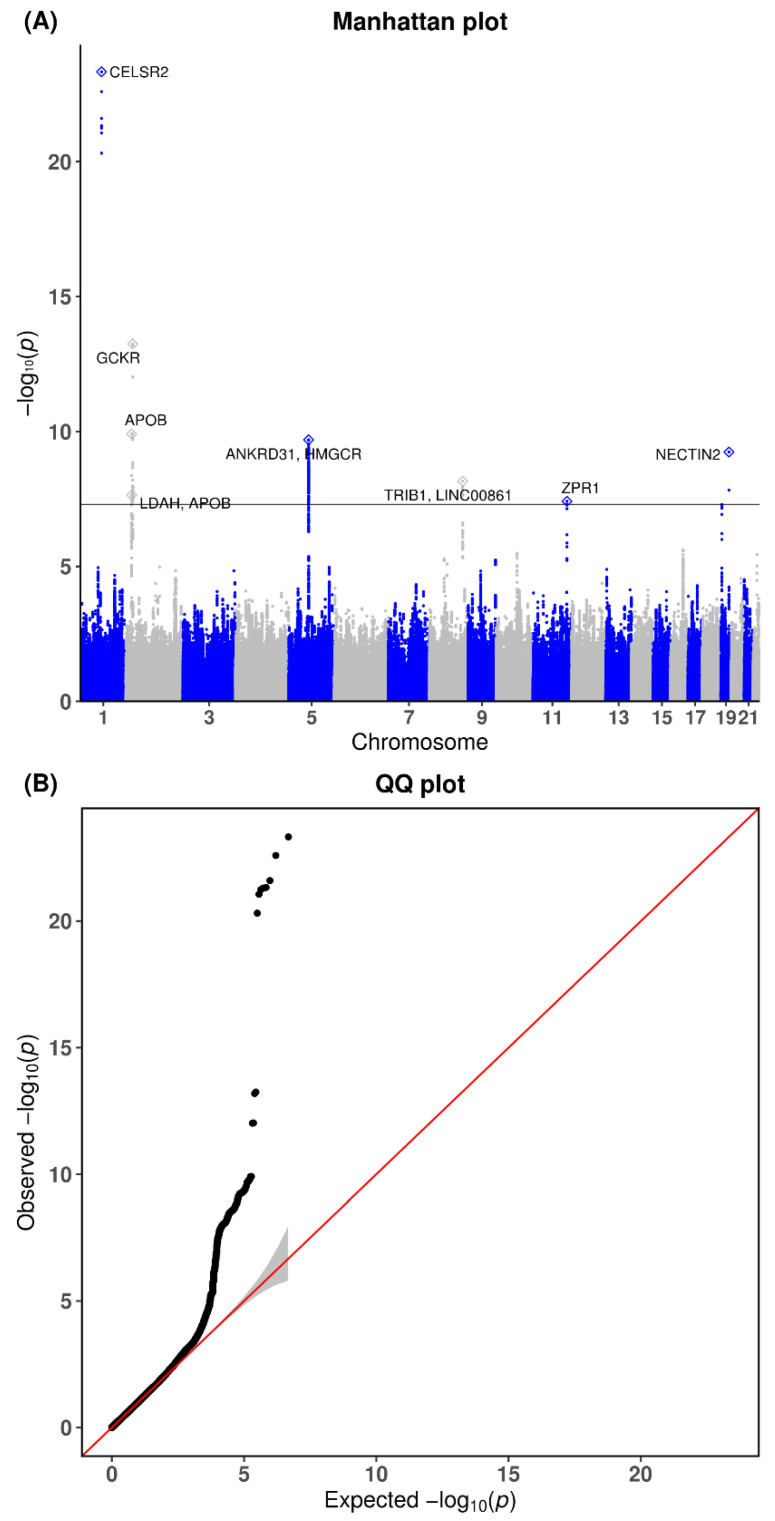
Genome-wide association of circulating ApoB by chromosome position, log10 *p*-value (Manhattan plot), and quantile-quantile plots for meta-analysis. (**A**) Manhattan plot of the *p*-values in the genome-wide association study meta-analysis for ApoB. (**B**) Quantile-quantile (Q-Q) plot showing expected vs. observed values [−log10 (*p*) values]. The expected line is shown in red, and confidence bands are shown in gray.

Overall, 3,417,851 SNPs from the KARE cohort and 3,505,163 SNPs from the CAVAS cohort were used. A total of 2,374,443 overlapping SNPs were selected as the final genetic markers for the GWAS meta-analysis of ApoA1, ApoB, and ApoB/ApoA1. Quantile–quantile (Q-Q) and Manhattan plots for ApoA1 are shown in Figure 2. The Q-Q plot revealed no evidence that the test statistics were inflated (λ = 1.024). The 16 genome-wide-significant variants for ApoA1 are listed in Table 2. Of them, the novel variants were rs11066280 (effect = −4.002, standard error [SE] = 0.424, *p* = 3.46 × $10^{-21},$ *HetPVal* = 0.8034) near HECT Domain E3 Ubiquitin Protein Ligase 4 (*HECTD4*), followed by rs1227162 (effect = −3.823, SE = 0.484, *p* = 2.98 × $10^{-15},$ *HetPVal* = 0.2643) near *Myosin Light Chain 2* (*MYL2*) and *Long Intergenic Non-Protein Coding RNA 1405* (*LINC01405*), and rs73216931 (effect = −2.059, SE = 0.353, *p* = 5.62 × $10^{-9},$ *HetPVal* = 0.6035) near *Lysine Methyltransferase 5A* (*KMT5A*). The Q-Q and Manhattan plots for ApoB are shown in Figure 3. The Q-Q plot revealed no evidence of any inflation of the test statistics (λ = 1.015). The GWAS meta-analysis for ApoB revealed 8 genome-wide-significant loci, all of which were previously reported (Table 3). The Q-Q and Manhattan plots for the ApoB/ApoA1 ratio are shown in Figure S1. The Q-Q plot revealed no evidence of test statistic inflation (λ = 1.016). The GWAS meta-analysis for ApoB/ApoA1 revealed 9 genome-wide-significant loci (Table S1).

### 3.3. Regional Analysis and Functional Annotation

**Figure 4:**
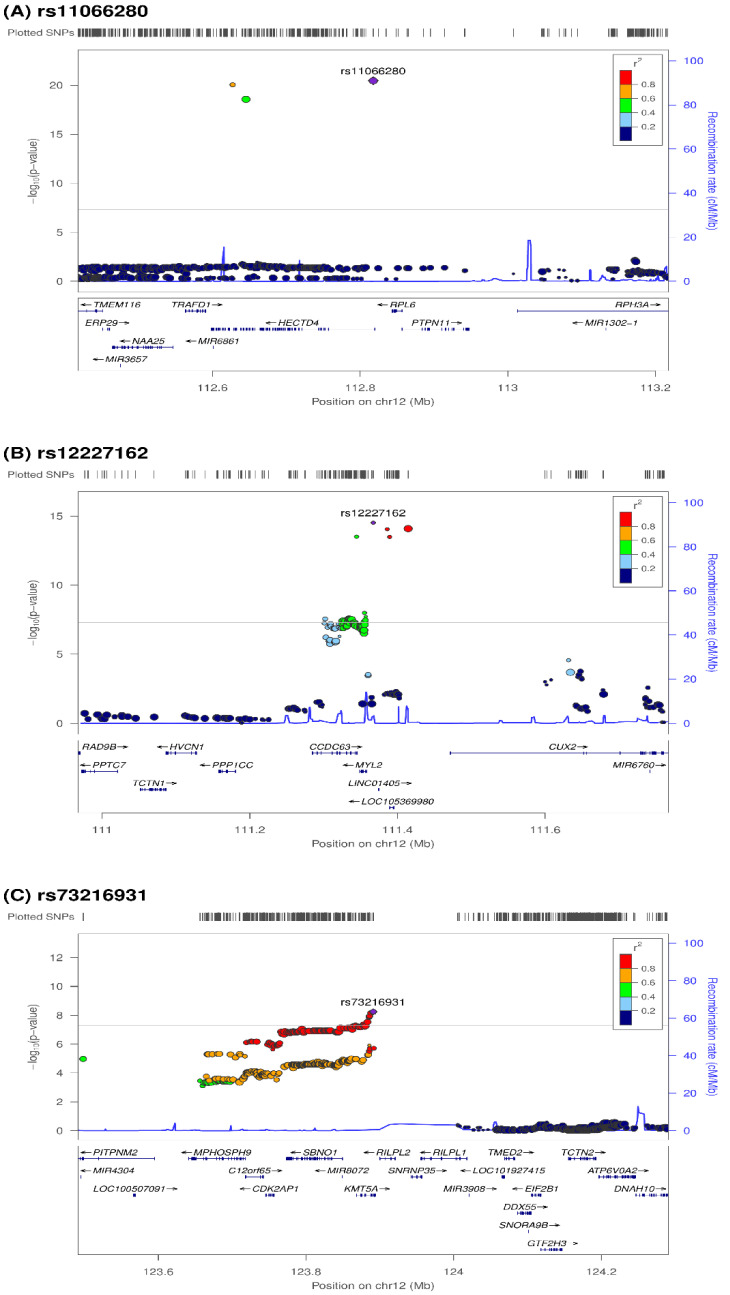
Regional plot of associated SNPs with genome-wide significance.

**Figure 5:**
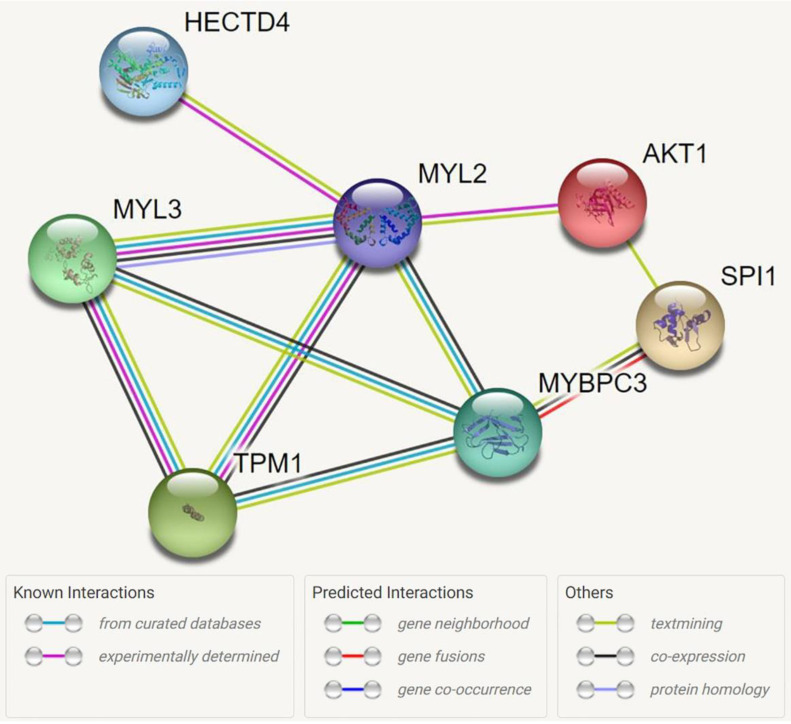
Network analysis of ApoA1.

For the genome-wide-significant novel phenotype variants of ApoA1, the regional plots with the lead SNPs are displayed in Figure 4. The eQTL analysis showed *KMT5A* expression in the pancreas and whole blood and high *MPHOSPH9* expression in the heart ventricles and atria (Table S2). In the DEG analysis with GSE53201, several genes, including *CCL20* (adjusted *p* =3.70 × $10^{-4}$, *p* = 1.29 × $10^{-8}$), *PTGS2* (adjusted *p* = 1.42 × $10^{-2}$, *p* = 9.83 × $10^{-7}$), and *TNIP3* (adjusted *p* = 1.90 × $10^{-2}$, *p* = 1.97 × $10^{-6}$) were expressed more in the ApoA1 treatment group than in the control group (Table 4). All of the 11 genes that were significant in ApoA1 treatment were also significant in another data set (naive HDL treatment and control) from GSE53201 (Table S3). The network analysis of *HECTD4* and *MYL2* revealed that the pathways related to *AKT1* were also related to the ApoA1 level (Figure 5). Nine biological processes (GO: 0030049, GO:0055010, GO:0060048, GO:0008015, GO:0006937, GO:1903522, GO:0006942, GO:0051146, GO:0008016), one molecular function (GO:0008307), and two cellular components (GO:0030017, GO:0016459) were associated with this network (Table 5). In addition, four KEGG pathways were related to cardiomyopathies (hsa05410, hsa05414, hsa04261, hsa04260, Table 5). With the exception of the novel loci identified in our investigation, subsequent GO and KEGG pathway analyses of the remaining significant loci related to ApoA1 and ApoB/ApoA1 showed a dyslipidemia-related function (Figure S2 and Table S4). However, GO analysis of ApoB-related genes, which was significant in our GWAS, revealed no significant GO terms.

### 3.4. Association between Vitamin D and ApoA1, ApoB, and ApoB/ApoA1

Vitamin D was positively associated with ApoA1 in the KARE cohort (β = 0.235, *p* < 0.001), CAVAS cohort (β = 0.447, *p* < 0.001), and combined set (β = 0.387, *p* < 0.001, Table 6). In addition, the ApoB/ApoA1 ratio was negatively associated with vitamin D levels in the KARE cohort (β = −0.002, *p* < 0.001), CAVAS cohort (β = −0.001, *p* < 0.001), and combined set (β = −0.002, *p* < 0.001). However, we found no clear evidence of an association between ApoB and vitamin D levels. Genetic variants associated with the ApoA1 and vitamin D levels had similar directions, implying that the ApoA1 and vitamin D levels might share common risk factors for CVDs (Table S5).

## 4. Discussion

Our study has demonstrated that three novel SNPs (rs11066280, rs1227162, and rs73216931) are linked to ApoA1 with GWAS significance. The functional analysis suggests that *HECTD4*, *MYL2*, and *KMT5A* are potential genes involved in the pathophysiology of ApoA1-related CVDs. Our GO and KEGG analyses revealed that *HECTD4* and *MYL2* are associated with cardiomyopathy, suggesting that the connection between *AKT1* and the ApoA1 level has a direct connection to CVDs. Furthermore, the findings of the study, with the exception of the novel loci, demonstrated the dyslipidemia-related functions of ApoA1 and ApoB/ApoA1-related genes. In addition, using regression analyses, we found that the vitamin D concentration was positively linked to the ApoA1 concentration, and we have summarized the genes connected to both vitamin D and ApoA1. Thus, a genomic analysis of the combined effects of ApoA1 and vitamin D on CVDs could uncover meaningful pathological mechanisms.

ApoA1 is a major component of HDL, which drives the reverse transport of cholesterol from extrahepatic tissue to the liver and plays an essential role in protecting the arteries [39]. Because ApoB is a risk factor for CVDs, we expected the ApoB/ApoA1 to provide cardiovascular information in the GWAS results. In this study, we confirmed several loci, including three novel genes related to ApoA1. Additionally, although no novel loci were identified for ApoB or ApoB/ApoA1, SNPs associated with them were found.

The functional analysis of our data for two new loci (*HECTD4* and *MYL2*) shows that they are associated with cardiomyopathy, suggesting that the association between *AKT1* and the ApoA1 level has a direct connection to CVDs. *AKT1* (protein kinase B) is a serin/threonine kinase, and ApoA1 enhances insulin-dependent and insulin-independent glucose uptake by skeletal muscle [40]. By activating downstream effectors and controlling the cell cycle transition, growth, and proliferation, PI3K/Akt signaling plays a vital role in regulating several physiological activities. This pathway has a role in the pathophysiology of various human ailments, including heart disease, by modulating cardiomyocyte growth and survival, angiogenic processes, and inflammatory responses [41]. Although a previous study showed that the PI3K/Akt pathway was not the main driver of HDL-mediated cell protection [42], our results indicate that it could nonetheless be involved. From this perspective, it is crucial to examine the causal relationships between CVDs and apolipoproteins. A previous observation analysis of ApoA1 and CVDs showed that ApoA1 was associated with a lower risk of CVDs. However, it was unrelated to the risk of CVD in that same study’s MR analysis [43]. Those results suggest that genetic evidence does not support a cardioprotective role for ApoA1. Several investigations of the connection between vitamin D and CVDs have been conducted [44,45], but few causal results are available. The observational and MR analyses of participants with a vitamin D deficiency (<25 nmol/L) in a recent study provided strong evidence for an inverse association between vitamin D deficiency and all-cause mortality during follow-up (odd ratio (OR):0.69 95CI:0.59–0.80, *p* < 0.001) and a non-significant inverse association between vitamin D deficiency and CVDs (OR 0.89 [0.76–1.04], *p* = 0.14) [46]. Due to the existence of many risk factors, such as hypertension and atherosclerosis, the ability to analyze the importance of a single metabolic phenotype relationship with CVDs is limited.

Our study found novel loci for ApoA1 (*HECTD4*, *MYL2*, and *KMT5A*) in a Korean cohort, and our results will carry more weight if replication studies in other groups of people support them. In this regard, we conducted phenome-wide association studies using the Common Metabolic Disease Knowledge Portal (https://hugeamp.org (accessed on29 June 2022 )). We found that SNPs such as rs11066280, rs1227162, and rs73216931 are related to metabolic conditions such as waist-hip ratio, lipid metabolism (HDL cholesterol and LDL cholesterol), type 2 diabetes, and body fat percentage in the European population (Table S6).

A major strength of our study is the inclusion of a relatively large study sample drawn from two Korean cohorts. However, this study also has a few limitations. First, we did not conduct an MR analysis for ApoA1 and CVDs, and because the KARE and CAVAS cohorts are both community-based cohorts, the number of patients with CVDs is small. To compensate for that limitation, additional research focusing on a heart-disease cohort is needed. Second, we could not perform DEGs analysis for ApoB or ApoB/ApoA1 because we could not find available data for differentially expressed genes (DEGs) analysis of ApoB or ApoB/ApoA1 in the Gene Expression Omnibus database. Third, due to a large number of missing values, we were unable to consider various confounding factors such as HOMA-IR and HOMA-beta cells in our regression analysis. ApoB and vitamin D correlated negatively in the KARE cohort but positively in the CAVAS cohort. Although we do not know the exact reason for this, it is possible that the pattern may be different, as the KARE is a cohort of chronic disease studies, and the CAVAS is a cohort of CVD studies. The relationship between vitamin D level and lipid metabolism warrants additional investigation. Fourth, we did not include vitamin D GWAS results in this study because a previous study had already conducted a vitamin D GWAS analysis in the KARE cohort [25]. Although our regression analysis revealed relationships between vitamin D and ApoA1 levels, along with genetic connections, causation was not demonstrated. To establish causation, experimental research using the genomic biomarkers identified in our work will be needed.

## 5. Conclusions

This study used a GWAS meta-analysis to identify three novel loci (rs11066280, rs1227162, and rs73216931) related to ApoA1. Furthermore, GO and KEGG analyses revealed that *HECTD4* and *MYL2* are associated with cardiomyopathy, suggesting that the association between *AKT1* and the ApoA1 level has a direct connection with CVDs. The analyses of associations between vitamin D and ApoA1 and vitamin D and ApoB/ApoA1 showed a real association in data from two Korean cohorts. In addition, shared genetic variants between ApoA1 and vitamin D levels could suggest that vitamin D plays an additive role in CVDs through an ApoA1-related pathway. Further study is needed to support the pathological mechanisms of ApoA1 in CVDs suggested by this genomic analysis.

## Figures and Tables

**Table 1:** Baseline characteristics of the study population.

	Total (*N* = 12,924)	KARE Cohort (*N* = 4938)	CAVAS Cohort (*N* = 7986)	*p*	Number of Missing KARE/CAVAS
Age (years)	58.05 ± 8.70	57.48 ± 8.46	58.40 ± 8.82	<0.001	0/0
Sex (male %)	5313 (41.1%)	2309 (46.8%)	3004 (37.6%)	<0.001	0/0
Weight (kg)	62.00 ± 9.95	62.90 ± 10.01	61.45 ± 9.88	<0.001	0/1
Height (cm)	158.78 ± 8.53	159.96 ± 8.77	158.06 ± 8.29	<0.001	0/1
Body mass index (kg/m^2^)	24.53 ± 3.03	24.53 ± 3.02	24.54 ± 3.04	0.935	0/1
Current smoking *n* (%)	1571 (14.7%)	804 (16.3%)	767 (13.4%)	<0.001	5/2251
Current drinking *n* (%)	5522 (42.8%)	2286 (46.3%)	3236 (40.6%)	<0.001	5/9
History of hypertension *n* (%)	2375 (18.4%)	218 (4.42%)	2157 (27%)	<0.001	6/7
History of diabetes *n* (%)	752 (5.82%)	90 (1.83%)	662 (8.3%)	<0.001	7/7
Systemic blood pressure (mmHg)	122.16 ± 17.17	118.34 ± 16.17	124.52 ± 17.34	<0.001	0/6
Diastolic blood pressure (mmHg)	78.19 ± 10.40	77.61 ± 9.65	78.56 ± 10.83	<0.001	0/6
Total cholesterol (mg/dL)	196.48 ± 34.94	195.74 ± 34.29	196.94 ± 35.32	0.056	0/1
LDL cholesterol (mg/dL)	123.91 ± 31.28	124.23 ± 30.68	123.71 ± 31.64	0.36	109/170
HDL cholesterol (mg/dL)	44.74 ± 10.76	44.10 ± 10.51	45.14 ± 10.90	<0.001	0/1
Triglycerides (mg/dL)	144.61 ± 93.73	141.68 ± 91.74	146.42 ± 94.90	0.005	0/3
Log Triglycerides	4.83 ± 0.52	4.80 ± 0.53	4.84 ± 0.52	<0.001	0/3
HbA1C (%)	5.75 ± 0.89	5.76 ± 0.90	5.70 ± 0.81	0.004	0/6471
Vitamin D (ng/mL)	21.25 ± 8.90	17.35 ± 7.22	23.67 ± 8.98	<0.001	0/20
Fasting glucose (mg/dL)	99.05 ± 25.27	100.34 ± 30.48	98.1 ± 20.6	<0.001	0/1
ApoA1 (mg/dL)	151.11 ± 26.26	145.24 ± 24.52	154.74 ± 26.64	<0.001	0/0
ApoB (mg/dL)	108.33 ± 24.90	106.79 ± 24.26	109.28 ± 25.24	<0.001	0/0
ApoB/ApoA1 ratio	0.74 ± 0.22	0.76 ± 0.22	0.73 ± 0.21	<0.001	0/0
HOMA-IR	2.28 ± 2.56	2.50 ± 3.18	1.98 ± 1.29	<0.001	0/4290
HOMA-beta cell	107.40 ± 80.43	110.26 ± 76.84	103.57 ± 84.86	<0.001	0/4290

**Table 2:** Results of GWAS meta-analysis of ApoA1 (leading SNPs).

Chr	SNP	Position	Allele	Independent Study					Meta-Analysis			Mapped Genes
				Cohort	Effect	SE	MAF	*p*	Effect	SE	*p*	
											(HetPVal)	
12	rs11066280	112817783	A/T	KARE	−4.123	0.646	0.17	1.89 × 10^−10^	−4.002	0.424	3.46 × 10^−21^	*HECTD4* (intronic)
				CAVAS	−3.91	0.561	0.16	3.45 × 10^−12^			(0.8034)	
15	rs16940212	58694020	T/G	KARE	3.37	0.513	0.34	5.79 × 10^−11^	3.09	0.336	3.62 × 10^−20^	*AQP9;LIPC* (intergenic)
				CAVAS	2.88	0.444	0.34	9.37 × 10^−11^			(0.4704)	
9	rs2740488	107661742	C/A	KARE	−4.192	0.558	0.26	6.59 × 10^−14^	3.341	0.369	1.49 × 10^−19^	*ABCA1* (intronic)
				CAVAS	−2.676	0.493	0.25	5.90 × 10^−8^			(0.0417)	
11	rs75198898	116649806	A/G	KARE	−5.302	0.931	0.07	1.31 × 10^−8^	−5.327	0.599	5.64 × 10^−19^	*ZPR1* (intronic)
				CAVAS	−5.345	0.782	0.08	8.54 × 10^−12^			(0.9718)	
15	rs6494007	58734645	T/C	KARE	3.196	0.572	0.25	2.44 × 10^−8^	3.197	0.37	5.17 × 10^−18^	*LIPC-AS1* (ncRNA_intronic)
				CAVAS	3.197	0.484	0.26	4.29 × 10^−11^			(0.9989)	
11	rs61905084	116610294	C/T	KARE	2.94	0.554	0.27	1.15 × 10^−7^	−3.03	0.36	4.32 × 10^−17^	*LINC02702;BUD13* (intergenic)
				CAVAS	3.096	0.475	0.26	7.49 × 10^−11^			(0.8307)	
8	rs35495249	19865509	A/C	KARE	2.97	0.596	0.21	6.33 × 10^−7^	3.088	0.389	1.93 × 10^−15^	*LPL;SLC18A1* (intergenic)
				CAVAS	3.175	0.513	0.21	6.27 × 10^−10^			(0.7942)	
12	rs12227162	111367244	T/C	KARE	−4.421	0.722	0.13	1.01 × 10^−9^	−3.823	0.484	2.98 × 10^−15^	*MYL2;LINC01405* (intergenic)
				CAVAS	−3.334	0.653	0.12	3.36 × 10^−7^			(0.2643)	
15	rs11858164	58742731	G/T	KARE	−2.2	0.558	0.26	8.06 × 10^−5^	2.64	0.362	3.06 × 10^−13^	*LIPC-AS1* (ncRNA_intronic)
				CAVAS	−2.962	0.476	0.26	5.26 × 10^−10^			(0.2987)	
7	rs146148222	80304855	G/T	KARE	5.099	1.115	0.05	4.88 × 10^−6^	−4.728	0.665	1.16 × 10^−12^	*CD36* (intronic)
				CAVAS	4.523	0.829	0.07	4.93 × 10^−8^			(0.6783)	
15	rs62001712	58653911	A/G	KARE	−2.043	0.551	0.28	2.08 × 10^−4^	−2.473	0.358	4.73 × 10^−12^	*AQP9;LIPC* (intergenic)
				CAVAS	−2.788	0.471	0.28	3.30 × 10^−9^			(0.3037)	
18	rs12458441	47122158	C/A	KARE	2.075	0.563	0.27	2.29 × 10^−4^	−2.361	0.363	7.42 × 10^−11^	*LIPG* (UTR3)
				CAVAS	2.565	0.474	0.28	6.57 × 10^−8^			(0.5055)	
16	rs1800775	56995236	C/A	KARE	−1.796	0.489	0.46	2.40 × 10^−4^	1.95	0.319	9.68 × 10^−10^	*CETP* (upstream)
				CAVAS	−2.065	0.421	0.45	9.57 × 10^−7^			(0.6766)	
15	rs539901	58674669	G/T	KARE	3.339	0.805	0.1	3.43 × 10^−5^	−3.113	0.525	2.95 × 10^−9^	*AQP9;LIPC* (intergenic)
				CAVAS	2.947	0.692	0.1	2.06 × 10^−5^			(0.7119)	
12	rs73216931	123891209	C/T	KARE	2.282	0.556	0.29	4.13 × 10^−5^	−2.059	0.353	5.62 × 10^−9^	*KMT5A* (intronic)
				CAVAS	1.908	0.458	0.3	3.08 × 10^−5^			(0.6035)	
11	rs1076485	116772441	C/T	KARE	−1.339	0.497	0.45	7.11 × 10^−3^	1.778	0.323	3.64 × 10^−8^	*SIK3* (intronic)
				CAVAS	−2.099	0.425	0.45	7.87 × 10^−7^			(0.2451)	

Chr, chromosome; SNP, single nucleotide polymorphism; MAF, minor allele frequency; SE, standard error; Mapped Genes from ANNOVAR; GWAS, genome-wide association study; KARE, Korean Association Resource; CAVAS, Cardiovascular Disease Association Study.

**Table 3:** Results of GWAS meta-analysis of ApoB (leading SNPs).

Chr	SNP	Position	Allele	Independent Study					Meta-Analysis			Mapped Genes
				Cohort	Effect	SE	MAF	*p*	Effect	SE	*p*	
											(HetPVal)	
1	rs611917	109815252	G/A	KARE	−7.6	1.057	0.05	7.66 × 10^−13^	6.643	0.657	4.69 × 10^−24^	*CELSR2* (intronic)
				CAVAS	−6.042	0.838	0.06	6.02 × 10^−13^			(0.2482)	
2	rs3817588	27731212	C/T	KARE	−2.961	0.516	0.32	9.86 × 10^−9^	2.45	0.326	5.71 × 10^−14^	*GCKR* (intronic)
				CAVAS	−2.11	0.421	0.32	5.51 × 10^−7^			(0.2011)	
2	rs11901649	21250223	A/G	KARE	3.038	0.826	0.09	2.38 × 10^−4^	3.32	0.516	1.24 × 10^−10^	*APOB* (intronic)
				CAVAS	3.501	0.661	0.1	1.20 × 10^−7^			(0.6617)	
5	rs10474433	74616843	C/T	KARE	2.396	0.518	0.33	3.87 × 10^−6^	−2.073	0.326	2.03 × 10^−10^	*ANKRD31;HMGCR* (intergenic)
				CAVAS	1.861	0.419	0.33	9.19 × 10^−6^			(0.4222)	
19	rs404935	45372794	A/G	KARE	2.2	0.716	0.14	2.14 × 10^−3^	2.799	0.452	5.78 × 10^−10^	*NECTIN2* (intronic)
				CAVAS	3.195	0.582	0.13	4.21 × 10^−8^			(0.281)	
8	rs2954029	126490972	A/T	KARE	2.163	0.483	0.44	7.76 × 10^−6^	1.767	0.305	6.99 × 10^−9^	*TRIB1;LINC00861* (intergenic)
				CAVAS	1.504	0.393	0.44	1.32 × 10^−4^			(0.2902)	
2	rs72788559	21195522	T/G	KARE	−2.938	0.711	0.13	3.62 × 10^−5^	−2.609	0.467	2.28 × 10^−8^	*LDAH;APOB* (intergenic)
				CAVAS	−2.359	0.619	0.12	1.39 × 10^−4^			(0.5389)	
11	rs75198898	116649806	A/G	KARE	3.078	0.916	0.07	7.81 × 10^−4^	3.132	0.57	3.87 × 10^−8^	*ZPR1* (intronic)
				CAVAS	3.166	0.728	0.08	1.38 × 10^−5^			(0.94)	

Chr, chromosome; SNP, single nucleotide polymorphism; MAF, minor allele frequency; SE, standard error; Mapped Genes from ANNOVAR; GWAS, genome-wide association study; KARE, Korean Association Resource; CAVAS, Cardiovascular Disease Association Study.

**Table 4:** Differentially expressed genes at the significant loci for ApoA1 in the Gene Expression Omnibus GSE53201 database.

Gene Symbol	Log FC	AveExpr	*p* Value	Adj *p* Value	B
*CCL20*	−0.841	0.354	1.29 × 10^−8^	3.70 × 10^−4^	6.470
*PTGS2*	−0.694	−0.058	9.83 × 10^−7^	1.42 × 10^−2^	4.253
*TNIP3*	−0.587	0.156	1.97 × 10^−6^	1.90 × 10^−2^	3.829
*SLC7A2*	−0.499	0.180	3.86 × 10^−6^	2.36 × 10^−2^	3.406
*VCAM1*	−0.626	0.302	4.09 × 10^−6^	2.36 × 10^−2^	3.368
*TNFAIP6*	−0.791	0.349	5.17 × 10^−6^	2.49 × 10^−2^	3.215
*CYP1A1*	0.861	−0.529	8.87 × 10^−6^	3.55 × 10^−2^	2.858
*DLL4*	−0.570	0.174	9.84 × 10^−6^	3.55 × 10^−2^	2.788
*SDF2L1*	0.594	0.012	1.17 × 10^−5^	3.75 × 10^−2^	2.670
*TNIP1*	−0.472	0.033	1.32 × 10^−5^	3.79 × 10^−2^	2.590
*TNFRSF9*	−0.516	0.025	1.69 × 10^−5^	4.42 × 10^−2^	2.419

Log FC = estimate of the log2-fold-change corresponding to the effect or contrast; AveExpr = average log2-expression for the probe over all arrays and channels; B = log-odds that the gene is differentially expressed.

**Table 5:** Gene Ontology and KEGG pathway analyses of novel loci-related genes associated with ApoA1.

Term ID	Term Description	Count in Network	Strength	FDR
**1. Biological process**				
GO:0030049	Muscle filament sliding	4/38	2.47	8.20 × 10^−6^
GO:0055010	Ventricular cardiac muscle tissue morphogenesis	4/48	2.37	8.20 × 10^−6^
GO:0060048	Cardiac muscle contraction	4/79	2.15	1.67 × 10^−5^
GO:0008015	Blood circulation	5/394	1.55	5.47 × 10^−5^
GO:0006937	Regulation of muscle contraction	4/162	1.84	1.00 × 10^−4^
GO:1903522	Regulation of blood circulation	4/292	1.58	7.30 × 10^−4^
GO:0006942	Regulation of striated muscle contraction	3/93	1.96	1.40 × 10^−3^
GO:0051146	Striated muscle cell differentiation	3/200	1.62	1.20 × 10^−2^
GO:0008016	Regulation of heart contraction	3/245	1.53	2.00 × 10^−2^
**2. Molecular function**				
GO:0008307	Structural constituent of muscle	4/43	2.41	3.29 × 10^−6^
**3. Cellular component**				
GO:0030017	Sarcomere	4/207	1.73	5.00 × 10^−4^
GO:0016459	Myosin complex	2/55	2.01	4.05 × 10^−2^
**4. KEGG**				
hsa05410	Hypertrophic cardiomyopathy	4/89	2.1	5.56 × 10^−6^
hsa05414	Dilated cardiomyopathy	4/95	2.07	5.56 × 10^−6^
hsa04261	Adrenergic signaling in cardiomyocytes	4/147	1.88	1.31 × 10^−5^
hsa04260	Cardiac muscle contraction	3/87	1.98	2.70 × 10^−4^

GO, Gene Ontology; KEGG, Kyoto Encyclopedia of Genes and Genomes; FDR, false discovery rate; Strength, Log10 (observed/expected): This measure describes how large the enrichment effect is. It is the ratio between the number of proteins in the network that are annotated with a term and the number of proteins that could be expected to be annotated with this term in a random network of the same size.

**Table 6:** Association between vitamin D and ApoA1, ApoB, and ApoB/ApoA1.

	KARE			CAVAS			KARE + CAVAS		
Outcome Variable	Beta	SE	*p* Value	Beta	SE	*p* Value	Beta	SE	*p* Value
ApoA1	0.235	0.056	<0.001	0.447	0.038	<0.001	0.387	0.031	<0.001
ApoB	−0.138	0.055	0.013	0.083	0.036	0.022	0.030	0.030	0.325
ApoB/ApoA1	−0.002	0.000	<0.001	−0.001	0.000	<0.001	−0.002	0.000	<0.001

Linear regression model with vitamin D as the predictor and ApoA1, ApoB, or ApoB/ApoA1 as the outcome after adjusting for age, sex, body mass index, and season of blood draw.

## Data Availability

GWAS dataset and epidemiological data for KoGES are available with the permission of the data access committee of the National Biobank of Korea (https://nih.go.kr/biobank/cmm/main/engMainPage.do (accessed on 27 April 2022)).

## Supplementary Materials

The following supporting information can be downloaded at: https://www.mdpi.com/article/10.3390/genes13091553/s1, Figure S1: Manhattan and quantile-quantile plots for ApoB/ApoA1 in meta-analysis. (A) Manhattan plot of the *p*-values in the genome-wide association study meta-analysis for ApoB/ApoA1. (B) Quantile-quantile (Q-Q) plot showing expected vs. observed values [−log10(*p*)values]. The expected line is shown in red, and confidence bands are shown in gray. Figure S2: Network analysis with a list of replicated genes (genes related to ApoA1, ApoB, and ApoB/ApoA1, that have been reported in other studies and were also found to be significant in this study). (A) ApoA1, (B) ApoB, and (C) ApoB/ApoA1. Table S1: Results of GWAS meta-analysis for ApoB/ApoA1 (leading SNPs). Table S2: cis-eQTLs of SNPs associated with ApoA1. Table S3: Differentially expressed genes for naive HDL in the Gene Expression Omnibus GSE53201 database (11 genes that were significantly differentially expressed for ApoA1). Table S4: Gene Ontology and KEGG pathway analyses using genes for ApoA1, ApoB and ApoB/ApoA1 are significant in our study and in previously reported studies. Table S5: List of SNPs Associated with Serum 25-Hydroxyvitamin D Concentration and Apolipoprotein A1 in Genome-Wide Analyses. Table S6: Links to phenome-wide association study (pheWAS) results using the “Common Metabolic Diseases Knowledge Portal” (https://hugeamp.org/ (accessed on 29 June 2022)).
