# Supplementary material for: A Genome-Wide Association Study of Genetic Variants of Apolipoprotein A1 Levels and Their Association with Vitamin D in Korean Cohorts
Source: Genes (Basel). 2022 Aug 29;13(9):1553. doi: 10.3390/genes13091553 (PMC9498618; doi:10.3390/genes13091553)
Supplement: Supplementary file 1 [file genes-13-01553-s001.zip › Supplementary_20220817.pdf]

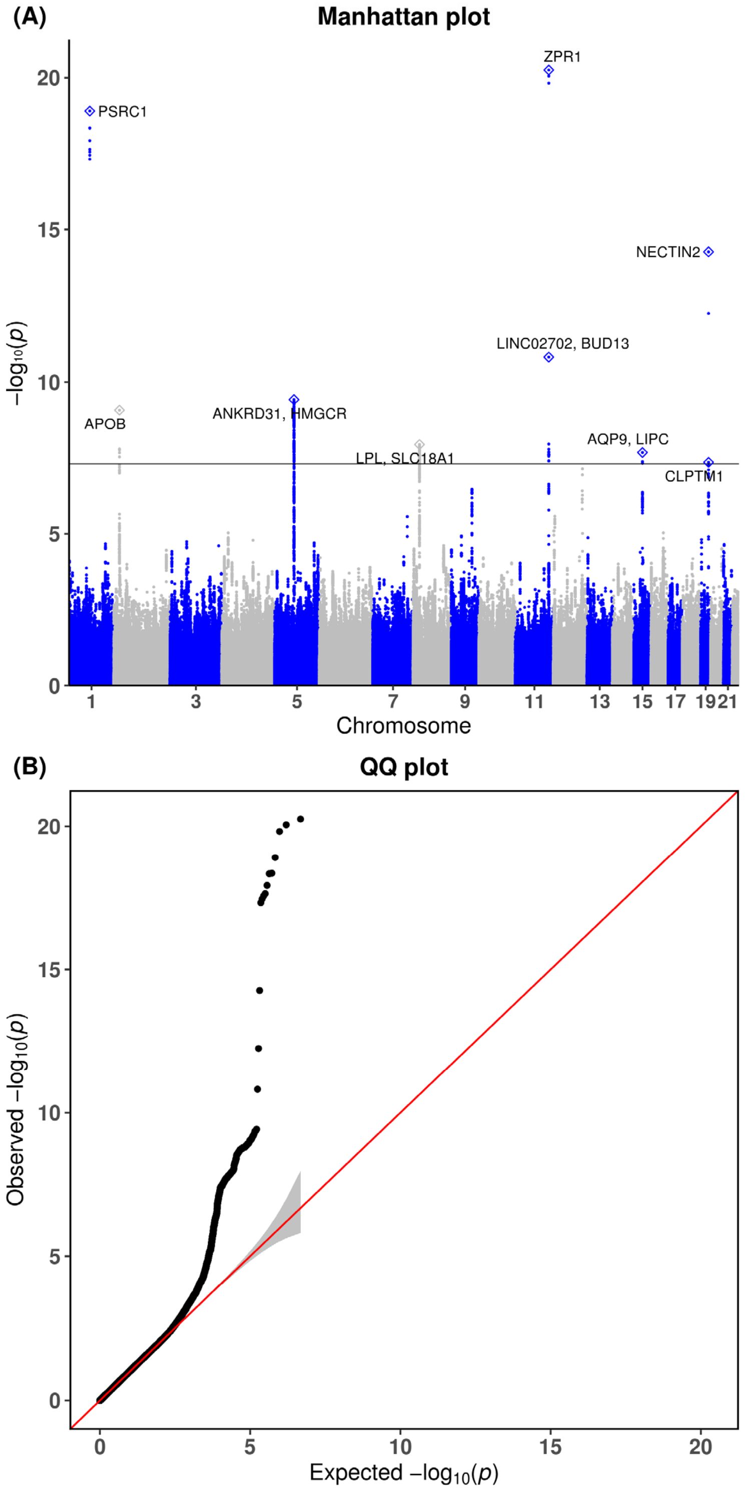

Supplementary Figure S1. Manhattan and quantile-quantile plots for ApoB/ApoA1 in meta-analysis. (A) Manhattan plot of the *P*-values in the genome-wide association study meta-analysis for ApoB/ApoA1. **(B)** Quantile-quantile (Q-Q) plot showing expected vs. observed values [$-\log_{10}(P)$values]. The expected line is shown in red, and confidence bands are shown in gray.

Abbreviations: QQ-plot, quantile-quantile plot; ApoA1, apolipoprotein A1; ApoB, apolipoprotein B

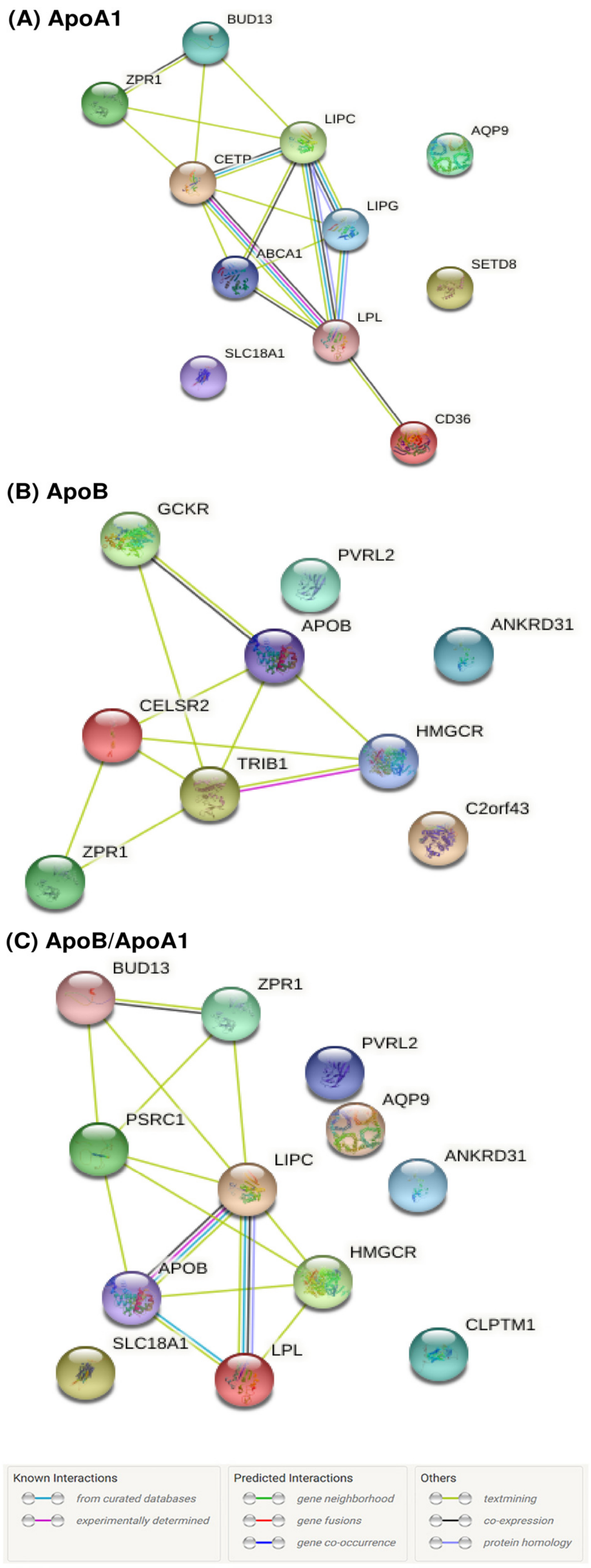

Figure S2: Network analysis with a list of replicated genes (genes related to ApoA1, ApoB and ApoB/ApoA1, that have been reported in other studies and were also found to be significant in this study). (A) ApoA1, (B) ApoB, and (C) ApoB/ApoA1.

Supplementary Table S1. Results of GWAS meta-analysis for ApoB/ApoA1 (leading SNPs)

					Independent study				Meta-analysis			
**Chr**	**SNP**	**Position**	**Allele**	**Cohort**	**Effect**	**SE**	**MAF**	***P***	**Effect**	**SE**	***P* (HetPVal)**	**Mapped Genes**
11	rs75198898	116649806	A/G	KARE	0.047	0.008	0.07	9.54×10^{-9}	0.046	0.005	5.57×10^{-21}	*ZPR1 (intronic)*
				CAVAS	0.046	0.006	0.08	1.16×10^{-13}			(0.9149)	
1	rs602633	109821511	T/G	KARE	-0.058	0.009	0.06	6.17×10^{-10}	-0.051	0.006	1.24×10^{-19}	*PSRC1 (downstream)*
				CAVAS	-0.047	0.007	0.06	2.68×10^{-11}			(0.3783)	
19	rs404935	45372794	A/G	KARE	0.027	0.006	0.14	2.35×10^{-5}	0.031	0.004	5.41×10^{-15}	*NECTIN2 (intronic)*
				CAVAS	0.033	0.005	0.13	4.15×10^{-11}			(0.4894)	
11	rs61905084	116610294	C/T	KARE	-0.023	0.005	0.27	1.68×10^{-6}	0.02	0.003	1.52×10^{-11}	*LINC02702;BUD13 (intergenic)*
				CAVAS	-0.018	0.004	0.26	1.45×10^{-6}			(0.3954)	
5	rs10056811	74605220	A/G	KARE	0.02	0.005	0.33	2.96×10^{-5}	0.018	0.003	3.77×10^{-10}	*ANKRD31;HMGCR (intergenic)*
				CAVAS	0.017	0.004	0.32	2.84×10^{-6}			(0.6595)	
2	rs11901649	21250223	A/G	KARE	0.024	0.007	0.09	1.50×10^{-3}	0.028	0.004	8.35×10^{-10}	*APOB (intronic)*
				CAVAS	0.03	0.006	0.1	1.23×10^{-7}			(0.5059)	
8	rs35237252	19870271	A/C	KARE	-0.023	0.005	0.21	1.88×10^{-5}	-0.019	0.003	1.16×10^{-8}	*LPL;SLC18A1 (intergenic)*
				CAVAS	-0.016	0.004	0.21	9.90×10^{-5}			(0.3254)	
15	rs16940212	58694020	T/G	KARE	-0.023	0.005	0.34	4.10×10^{-7}	-0.016	0.003	2.13×10^{-8}	*AQP9;LIPC (intergenic)*
				CAVAS	-0.011	0.004	0.34	1.62×10^{-3}			(0.0385)	
19	rs57204168	45496776	G/A	KARE	-0.012	0.004	0.47	5.72×10^{-3}	0.015	0.003	4.48×10^{-8}	*CLPTM1 (downstream)*
				CAVAS	-0.016	0.003	0.46	1.88×10^{-6}			(0.4991)	

Chr, chromosome; SNP, single nucleotide polymorphism; MAF, minor allele frequency; SE, standard error; Mapped Genes from ANNOVAR; GWAS, genome-wide association study; KARE, Korean Association Resource; CAVAS, Cardiovascular Disease Association Study

Supplementary Table S2. cis-eQTLs of SNPs associated with ApoA1

Gene Symbol	P-Value	NES	Tissue
KMT5A	4.7×10^{-20}	0.51	Pancreas
KMT5A	3.6×10^{-15}	-0.22	Whole Blood
ABCB9	8.3×10^{-15}	-0.48	Liver
ABCB9	1.5×10^{-14}	-0.34	Heart - Left Ventricle
KMT5A	5.8×10^{-13}	0.49	Brain - Cerebellum
C12orf65	5.1×10^{-12}	0.22	Adipose - Subcutaneous
ABCB9	2.4×10^{-11}	-0.19	Artery - Tibial
RP11-282O18.3	4.0×10^{-11}	0.26	Adipose - Visceral (Omentum)
CDK2AP1	5.8×10^{-11}	0.2	Adipose - Subcutaneous
PITPNM2	4.6×10^{-10}	0.39	Brain - Cerebellar Hemisphere
C12orf65	3.6×10^{-9}	0.18	Adipose - Visceral (Omentum)
CDK2AP1	7.9×10^{-9}	0.18	Adipose - Visceral (Omentum)
ARL6IP4	1.9×10^{-8}	0.21	Pancreas
PITPNM2	2.3×10^{-8}	0.33	Brain - Cerebellum
C12orf65	5.4×10^{-8}	0.22	Artery - Aorta
ARL6IP4	9.8×10^{-8}	0.082	Whole Blood
ABCB9	1.0×10^{-7}	-0.26	Heart - Atrial Appendage
RP11-282O18.3	1.8×10^{-7}	0.27	Artery - Aorta
CDK2AP1	4.3×10^{-7}	0.24	Pancreas
RP11-282O18.3	7.1×10^{-7}	0.41	Brain - Cerebellar Hemisphere
ARL6IP4	7.4×10^{-7}	0.12	Adipose - Visceral (Omentum)
RP11-282O18.3	7.8×10^{-7}	-0.26	Whole Blood
ABCB9	1.0×10^{-6}	-0.13	Whole Blood
OGFOD2	3.4×10^{-6}	0.19	Pancreas
RP11-282O18.3	3.5×10^{-6}	0.25	Heart - Atrial Appendage
MPHOSPH9	5.9×10^{-6}	-0.14	Artery - Tibial
RP11-282O18.3	6.4×10^{-6}	0.17	Artery - Tibial
RP11-282O18.3	8.9×10^{-6}	0.17	Adipose - Subcutaneous
ARL6IP4	1.1×10^{-5}	0.11	Heart - Atrial Appendage
MPHOSPH9	1.3×10^{-5}	-0.11	Heart - Left Ventricle
KMT5A	2.4×10^{-5}	0.19	Brain - Caudate (basal ganglia)
C12orf65	2.9×10^{-5}	-0.14	Whole Blood
MPHOSPH9	3.5×10^{-5}	-0.15	Heart - Atrial Appendage
C12orf65	7.7×10^{-5}	0.14	Artery - Tibial
RP11-282O18.3	7.9×10^{-5}	0.31	Brain - Caudate (basal ganglia)
SNRNP35	1.0×10^{-4}	-0.066	Whole Blood
ABCB9	1.1×10^{-4}	-0.17	Pancreas
CDK2AP1	2.0×10^{-4}	0.08	Artery - Tibial

Supplementary Table S3. Differentially expressed genes for naive HDL in the Gene Expression Omnibus GSE53201 database (11 genes which were significantly differentially expressed for ApoA1)

Gene symbol	Log FC	AveExpr	P value	Adj P value	B
CCL20	-1.346	0.050	4.50×10^{-7}	2.73×10^{-4}	6.795
PTGS2	-0.286	0.101	4.30×10^{-3}	4.76×10^{-2}	-2.187
TNIP3	-0.837	-0.015	1.72×10^{-9}	7.07×10^{-6}	11.731
SLC7A2	-0.376	0.196	2.36×10^{-5}	2.02×10^{-3}	2.966
VCAM1	-1.259	-0.063	5.62×10^{-8}	8.52×10^{-5}	8.719
TNFAIP6	-1.144	0.121	4.23×10^{-7}	2.67×10^{-4}	6.853
CYP1A1	2.723	0.305	8.26×10^{-12}	2.38×10^{-7}	15.608
DLL4	-0.564	0.131	2.86×10^{-5}	2.26×10^{-3}	2.775
SDF2L1	0.345	-0.125	5.53×10^{-5}	3.49×10^{-3}	2.123
TNIP1	-0.506	-0.029	2.61×10^{-6}	6.28×10^{-4}	5.117
TNFRSF9	-0.857	-0.190	6.17×10^{-7}	3.02×10^{-4}	6.496

Log FC=estimate of the log2-fold-change corresponding to the effect or contrast; AveExpr=average log2-expression for the probe over all arrays and channels; B= log-odds that the gene is differentially expressed

Supplementary Table S4. Gene Ontology and KEGG pathway analyses using genes for ApoA1, ApoB and ApoB/ApoA1 that are significant in our study and in previously reported studies

ApoA1

Term ID	Term description	Count in network	Strength	FDR
	**1. Biological process**			
GO:0015850	Organic hydroxy compound transport	7/145	1.93	6.11×10^{-9}
GO:0097006	Regulation of plasma lipoprotein particle levels	6/75	2.15	1.21×10^{-8}
GO:0071827	Plasma lipoprotein particle organization	5/47	2.28	2.12×10^{-7}
GO:0030301	Cholesterol transport	5/54	2.22	2.45×10^{-7}
GO:0043691	Reverse cholesterol transport	4/17	2.62	6.54×10^{-7}
GO:0042632	Cholesterol homeostasis	5/85	2.02	1.33×10^{-6}
GO:1905954	Positive regulation of lipid localization	5/93	1.98	1.64×10^{-6}
GO:0010743	Regulation of macrophage derived foam cell differentiation	4/31	2.36	2.90×10^{-6}
GO:0034369	Plasma lipoprotein particle remodeling	4/30	2.38	2.90×10^{-6}
GO:0006869	Lipid transport	6/296	1.56	4.76×10^{-6}
GO:0034372	Very-low-density lipoprotein particle remodeling	3/9	2.77	1.96×10^{-5}
GO:0055091	Phospholipid homeostasis	3/13	2.61	4.73×10^{-5}
GO:0010885	Regulation of cholesterol storage	3/16	2.52	7.43×10^{-5}
GO:0034375	High-density lipoprotein particle remodeling	3/18	2.47	9.75×10^{-5}
GO:0071404	Cellular response to low-density lipoprotein particle stimulus	3/19	2.45	1.10×10^{-4}
GO:0032376	Positive regulation of cholesterol transport	3/28	2.28	2.90×10^{-4}
GO:0048878	Chemical homeostasis	7/1124	1.04	2.90×10^{-4}
GO:0070328	Triglyceride homeostasis	3/35	2.18	4.70×10^{-4}
GO:0055096	Low-density lipoprotein particle mediated signaling	2/3	3.07	1.20×10^{-3}
GO:0034197	Triglyceride transport	2/5	2.85	2.00×10^{-3}
GO:0006641	Triglyceride metabolic process	3/69	1.89	2.50×10^{-3}
GO:0006820	Anion transport	5/593	1.18	3.10×10^{-3}
GO:0010886	Positive regulation of cholesterol storage	2/7	2.71	3.10×10^{-3}
GO:0015837	Amine transport	2/8	2.65	3.70×10^{-3}
GO:0006629	Lipid metabolic process	6/1190	0.95	5.10×10^{-3}
GO:0010745	Negative regulation of macrophage derived foam cell differentiation	2/13	2.44	7.90×10^{-3}
GO:0006644	Phospholipid metabolic process	4/373	1.28	9.70×10^{-3}
GO:0008203	Cholesterol metabolic process	3/120	1.65	9.70×10^{-3}

GO:0032611	interleukin-1 beta production	2/15	2.38	9.70×10^{-3}
GO:0034374	Low-density lipoprotein particle remodeling	2/15	2.38	9.70×10^{-3}
GO:0010744	Positive regulation of macrophage derived foam cell differentiation	2/18	2.3	1.20×10^{-2}
GO:0051004	Regulation of lipoprotein lipase activity	2/22	2.21	1.69×10^{-2}
GO:0019433	Triglyceride catabolic process	2/23	2.19	1.80×10^{-2}
GO:0051050	Positive regulation of transport	5/923	0.98	1.80×10^{-2}
GO:0007584	Response to nutrient	3/160	1.52	1.82×10^{-2}
GO:0015711	Organic anion transport	4/465	1.18	1.88×10^{-2}
GO:0044255	Cellular lipid metabolic process	5/939	0.98	1.88×10^{-2}
GO:0010874	Regulation of cholesterol efflux	2/30	2.07	2.71×10^{-2}
GO:0044242	Cellular lipid catabolic process	3/196	1.43	3.04×10^{-2}
GO:0034381	Plasma lipoprotein particle clearance	2/40	1.95	4.42×10^{-2}
GO:0009395	Phospholipid catabolic process	2/41	1.94	4.57×10^{-2}
GO:0006911	Phagocytosis, engulfment	2/43	1.92	4.87×10^{-2}
	**2. Molecular function**			
GO:0071813	Lipoprotein particle binding	4/29	2.39	7.09×10^{-6}
GO:0008970	Phospholipase a1 activity	3/12	2.65	4.06×10^{-5}
GO:0052739	Phosphatidylserine 1-acylhydrolase activity	3/11	2.69	4.06×10^{-5}
GO:0052740	1-acyl-2-lysophosphatidylserine acylhydrolase activity	3/10	2.73	4.06×10^{-5}
GO:0034185	Apolipoprotein binding	3/18	2.47	9.42×10^{-5}
GO:0004806	Triglyceride lipase activity	3/24	2.35	1.80×10^{-4}
GO:0004465	Lipoprotein lipase activity	2/2	3.25	7.00×10^{-4}
GO:0017129	Triglyceride binding	2/2	3.25	7.00×10^{-4}
GO:0008035	High-density lipoprotein particle binding	2/11	2.51	7.20×10^{-3}
GO:0005319	Lipid transporter activity	3/132	1.61	1.22×10^{-2}
GO:0030169	Low-density lipoprotein particle binding	2/17	2.32	1.22×10^{-2}
GO:0120020	Cholesterol transfer activity	2/21	2.23	1.46×10^{-2}
GO:0008201	Heparin binding	3/172	1.49	1.98×10^{-2}
GO:0031210	Phosphatidylcholine binding	2/28	2.1	2.11×10^{-2}
GO:0005215	Transporter activity	5/1181	0.88	3.87×10^{-2}
GO:0005548	Phospholipid transporter activity	2/46	1.89	4.13×10^{-2}
GO:0015485	Cholesterol binding	2/49	1.86	4.49×10^{-2}
GO:1901618	Organic hydroxy compound transmembrane transporter activity	2/49	1.86	4.49×10^{-2}
GO:0042887	Amide transmembrane transporter activity	2/52	1.84	4.51×10^{-2}

	**3. Cellular component**			
GO:0034358	Plasma lipoprotein particle	3/38	2.15	2.40×10^{-3}
	**4. KEGG**			
hsa04979	Cholesterol metabolism	6/48	2.35	5.08×10^{-11}
hsa00561	Glycerolipid metabolism	3/59	1.96	8.20×10^{-4}
hsa04975	Fat digestion and absorption	2/41	1.94	2.87×10^{-2}

ApoB/ApoA1

**Term ID**	**Term description**	**Count in network**	**Strength**	**FDR**
	**1. Biological process**			
GO:0034370	Triglyceride-rich lipoprotein particle remodeling	3/13	2.58	1.30×10^{-3}
GO:0019433	Triglyceride catabolic process	3/23	2.33	2.90×10^{-3}
GO:0015850	Organic hydroxy compound transport	4/145	1.65	4.10×10^{-3}
GO:0010886	Positive regulation of cholesterol storage	2/7	2.67	1.22×10^{-2}
GO:0015837	Amine transport	2/8	2.61	1.32×10^{-2}
GO:0034382	Chylomicron remnant clearance	2/8	2.61	1.32×10^{-2}
GO:0034371	Chylomicron remodeling	2/9	2.56	1.34×10^{-2}
GO:0034372	Very-low-density lipoprotein particle remodeling	2/9	2.56	1.34×10^{-2}
GO:0042632	Cholesterol homeostasis	3/85	1.76	1.34×10^{-2}
GO:0034374	Low-density lipoprotein particle remodeling	2/15	2.34	2.49×10^{-2}
GO:0008203	Cholesterol metabolic process	3/120	1.61	2.63×10^{-2}
GO:0010744	Positive regulation of macrophage derived foam cell differentiation	2/18	2.26	3.09×10^{-2}
GO:0006720	Isoprenoid metabolic process	3/142	1.54	3.57×10^{-2}
GO:0051004	Regulation of lipoprotein lipase activity	2/22	2.17	3.79×10^{-2}
	**2. Cellular component**			
GO:0034358	Plasma lipoprotein particle	3/38	2.11	3.10×10^{-3}
GO:0042627	Chylomicron	2/13	2.4	1.53×10^{-2}
GO:0034361	Very-low-density lipoprotein particle	2/20	2.21	2.68×10^{-2}
GO:0034364	High-density lipoprotein particle	2/29	2.05	3.85×10^{-2}
	**3. KEGG**			
hsa04979	Cholesterol metabolism	3/48	2.01	1.20×10^{-3}

ApoB-related genes had no significant GO terms.
GO, Gene Ontology; KEGG, Kyoto Encyclopedia of Genes and Genomes; FDR, false discovery rate; Strength, Log10(observed / expected): This measure describes how large the enrichment effect is. It is the ratio between the number of proteins in the network

that are annotated with a term and the number of proteins that could be expected to be annotated with this term in a random network of the same size.

Supplementary Table S5. List of SNPs Associated with Serum 25-Hydroxyvitamin D Concentration and Apolipoprotein A1 in Genome-Wide Analyses

						GWAS catalog						Our study			
						Vitamin D			ApoA1			ApoA1			
Chr	Position	SNP	Ref	Alt	Mapped Genes	Beta	95% CI	p value	Beta	95% CI	p-value	Cohort	Beta	SE	p-value
2	27730940	rs1260326	C	T	*GCKR*	0.022 unit decrease	[0.018-0.026]	4×10^{-28}	0.025843 unit increase	[0.022-0.03]	4×10^{-38}	CAVAS	1.082	0.4189	9.80×10^{-3}
2	27741237	rs780094	C	T	*GCKR*	0.0199 unit decrease	[0.015-0.025]	3×10^{-15}	0.0289 unit increase	[0.024-0.034]	3×10^{-29}	KARE + CAVAS	1.3952	0.3175	1.11×10^{-5}
2	211540507	rs1047891	C	A	*CPS1*	0.014168 unit decrease	[0.01-0.018]	1×10^{-11}	0.025611 unit decrease	[0.021-0.03]	5×10^{-34}	CAVAS	-1.05	0.5401	0.0519
11	117100029	rs4938362	G	A	*PCSK7*	0.0203 unit decrease	[0.014-0.027]	2×10^{-9}	0.0404 unit increase	[0.034-0.047]	7×10^{-32}	CAVAS	0.4671	0.6689	0.485
16	56995236	rs1800775	C	A	*CETP, HERPUD1*	0.016619 unit decrease	[0.013-0.02]	2×10^{-17}	-	-	3×10^{-6}	KARE + CAVAS	1.9503	0.319	9.68×10^{-10}
16	57002732	rs9939224	G	T	*CETP*	0.0199 unit increase	[0.014-0.026]	3×10^{-11}	0.1925 unit decrease	[0.19-0.2]	3×10^{-878}	CAVAS	-2.903	0.7147	4.87×10^{-5}
19	45412079	rs7412	C	T	*APOE*	0.033 unit decrease	[0.025-0.041]	4×10^{-20}	0.1291 unit increase	[0.12-0.14]	6×10^{-155}	CAVAS	4.795	0.869	3.43×10^{-8}
19	45413233	rs1065853	G	T	*APOE, APOC1*	0.027397 unit increase	[0.02-0.035]	8×10^{-14}	0.0915419 unit increase	[0.078-0.105]	2×10^{-39}	CAVAS	4.786	0.8697	3.73×10^{-8}
19	45429543	rs71352239	C	T	*APOC1, APOC1P1*	0.0164 unit decrease	[0.011-0.022]	3×10^{-9}	0.0192 unit decrease	[0.014-0.025]	1×10^{-11}	CAVAS	-0.3534	0.5342	0.5083
1	55505647	rs11591147	T	G	*PCSK9*	0.046 unit increase	[0.032-0.06]	4×10^{-10}	0.0532417 unit decrease	[0.039-0.068]	1×10^{-12}				
11	116710968	rs613808	G	A	*APOA1-AS*	0.027 unit decrease	[0.023-0.031]	1×10^{-33}	0.0849428 unit increase	[0.081-0.089]	7×10^{-331}				
11	116762829	rs75158858	G	A	*SIK3*	0.0289 unit decrease	[0.021-0.037]	3×10^{-13}	0.0721 unit increase	[0.064-0.08]	3×10^{-71}				
15	58689187	rs11855284	T	C	*ALDH1A2*	0.0303 unit decrease	[0.024-0.036]	7×10^{-23}	0.1252 unit increase	[0.12-0.13]	2×10^{-349}				
19	11202306	rs6511720	G	T	*LDLR*	0.0274 unit increase	[0.02-0.035]	2×10^{-13}	0.0271 unit increase	[0.019-0.035]	2×10^{-12}				
19	48376995	rs212100	C	T	*SULT2A1*	0.066 unit increase	[0.06-0.072]	1×10^{-135}	0.0149029 unit decrease	[0.0097-0.0201]	2×10^{-8}				
4	72615362	rs11723621	G	A	*GC*	1.28 unit decrease	[1.035-1.525]	2×10^{-24}				KARE + CAVAS	0.1469	0.3493	0.6742
4	72618334	rs7041	C	A	*GC*	0.79 unit decrease	[0.533-1.047]	2×10^{-9}				CAVAS	0.1017	0.4759	0.8308
11	14784110	rs11023332	G	C	*PDE3B*	0.77 unit increase	[0.543-0.997]	3×10^{-11}				KARE	0.6911	0.5015	0.1682
11	71132868	rs12803256	G	A	*ACTE1P*	0.65 unit decrease	[0.418-0.882]	4×10^{-8}				KARE + CAVAS	-0.3835	0.338	0.2566

Chr, chromosome; SNP, single nucleotide polymorphism; Ref, reference allele; Alt, alterative allele; Mapped Genes from ANNOVAR; CI, confidence interval; SE, standard error; GWAS, genome-wide association study; KARE, Korean Association Resource; CAVAS, Cardiovascular Disease Association Study

Supplementary Table S6. Links to phenome-wide association study (pheWAS) results using the "Common Metabolic Diseases Knowledge Portal" (https://hugeamp.org/).

PheWAS results for significant SNPs (rs12227162) in ApoA1
(https://hugeamp.org/variant.html?variant=rs12227162)
PheWAS results for significant SNPs (rs73216931) in ApoA1
(https://hugeamp.org/variant.html?variant=rs73216931)
PheWAS results for significant SNPs (rs11066280) in ApoA1
(https://hugeamp.org/variant.html?variant=rs11066280)
